# Fluorescent hiPSC-derived MYH6-mScarlet cardiomyocytes for real-time tracking, imaging, and cardiotoxicity assays

**DOI:** 10.1007/s10565-022-09742-0

**Published:** 2022-07-23

**Authors:** Reeja Maria Cherian, Chandra Prajapati, Kirsi Penttinen, Martta Häkli, Janne T. Koivisto, Mari Pekkanen-Mattila, Katriina Aalto-Setälä

**Affiliations:** 1 Heart Group, Faculty of Medicine and Health Technology, Tampere University, Tampere, Finland; 2 Biomaterials and Tissue Engineering Group, Faculty of Medicine and Health Technology, Tampere University, Tampere, Finland; 3 Division of Pathology, Department of Laboratory Medicine, Karolinska Institutet, Stockholm, Sweden; 4 Heart Hospital, Tampere University Hospital, Tampere, Finland

**Keywords:** Cardiac reporter, Drug toxicity screening, Biomaterial tracking and cytocompatibility tool, 2D/3D cardiotoxicity testing

## Abstract

Human induced pluripotent stem cell derived cardiomyocytes (hiPSC-CMs) hold great potential in the cardiovascular field for human disease modeling, drug development, and regenerative medicine. However, multiple hurdles still exist for the effective utilization of hiPSC-CMs as a human-based experimental platform that can be an alternative to the current animal models. To further expand their potential as a research tool and bridge the translational gap, we have generated a cardiac-specific hiPSC reporter line that differentiates into fluorescent CMs using CRISPR-Cas9 genome editing technology. The CMs illuminated with the mScarlet fluorescence enable their non-invasive continuous tracking and functional cellular phenotyping, offering a real-time 2D/3D imaging platform. Utilizing the reporter CMs, we developed an imaging-based cardiotoxicity screening system that can monitor distinct drug-induced structural toxicity and CM viability in real time. The reporter fluorescence enabled visualization of sarcomeric disarray and displayed a drug dose–dependent decrease in its fluorescence. The study also has demonstrated the reporter CMs as a biomaterial cytocompatibility analysis tool that can monitor dynamic cell behavior and maturity of hiPSC-CMs cultured in various biomaterial scaffolds. This versatile cardiac imaging tool that enables real time tracking and high-resolution imaging of CMs has significant potential in disease modeling, drug screening, and toxicology testing.

**Graphical abstract:**

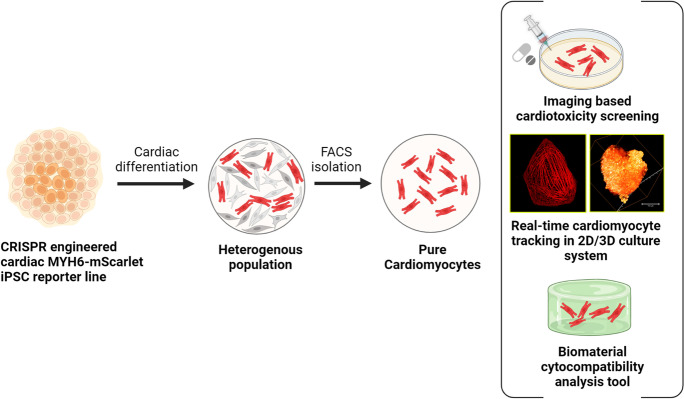

**Supplementary Information:**

The online version contains supplementary material available at 10.1007/s10565-022-09742-0.

## Introduction

Depletion of functional CMs is a hallmark characteristic of various cardiac diseases, e.g., heart failure, myocardial ischemia, and infarction (Chiong et al. 2011; Whelan et al. 2010). Due to the inadequate cardiomyocyte renewal in the heart and limited capacity for regeneration and repair, stem cell–based cardiac repair holds great potential to replace the dead myocardium and restore the function of the diseased heart (Braunwald 2018; Liew et al. 2020). HiPSC-CMs represent the ideal cell source for cardiac regeneration and can recapitulate genetically acquired cardiac disease phenotypes to evaluate novel therapeutics (Duelen and Sampaolesi 2017; Mazzola and di Pasquale 2020; Takahashi and Yamanaka 2006). However, utilization of these cells to their full potential is limited due to their immaturity and difference in the functional and electrophysiological characteristics compared to adult CMs.

HiPSCs differentiate into a heterogeneous cardiovascular population, including CMs, smooth muscle cells, fibroblasts, and endothelial cells with varying abundance depending on the cell line of origin and culture conditions (Moretti et al. 2006; Ojala et al. 2012). The presence of different cell types is important for the maturation of CMs; however, the varying amounts of contaminating cells produced during each differentiation process could affect the analysis of certain functional parameters of CMs, like drug response (Chen et al. 2014; Fuerstenau-Sharp et al. 2015). Hence, it is important to identify and derive pure CM populations in sufficient quantity and maturity to establish advanced cardiac culture systems with a balanced cocktail of cells that can mimic the heart more accurately. However, disassociation techniques routinely utilized to enrich the CMs from the differentiation are laborious, time consuming, and expensive. Advances in the CRISPR-Cas9 genome editing technology have allowed us the easy target selection of any cell type by the fluorescent tagging of an endogenous protein of interest (den Hartogh and Passier 2016; Sharma et al. 2018; Sontayananon et al. 2020). These fluorescent reporters can also be used to non-invasively study intracellular protein localization, function, and dynamics. Majority of the cardiac reporter lines so far generated are in human embryonic stem cell line (hESC) (den Hartogh et al. 2015; Elliott et al. 2011; Ghazizadeh et al. 2022; Ritner et al. 2011; Schwach et al. 2017; Tsai et al. 2020), and only a few of them are reported in hiPSC that can faithfully recapitulate the endogenous activation of the selected cardiac protein (Chirikian et al. 2021; Garreta et al. 2016; Zhang et al. 2019).

Here, we report the generation of a CRISPR engineered cardiac MYH6-mScarlet hiPSC reporter line that allows the real-time monitoring of cardiac differentiation and can track the fate and function of the differentiated CMs. The hiPSC-CMs illuminated with the mScarlet fluorescent protein of record brightness and photostability provide a means of non-invasive, continuous, and real-time tracking of the CM location, behavior, and functional status over time in both 2D and 3D culture environments. They enable a cardiac imaging system that provides a quantitative measure for comparing the efficacy of various CM differentiation protocols, studies the effect of different biomaterial scaffolds on the cell behavior, and monitors the cardiotoxic effects of drugs. Using the reporter line, the impact of different biomaterials on CM morphology, alignment, and maturation was analyzed by live cell imaging and gene expression profiling of cardiac-specific genes. We investigated the cytocompatibility of commonly used extracellular matrix protein–based culture substrates including gelatin and laminin, a textile-based scaffold polyethylene terephthalate (PET), and hydrazone crosslinked hydrogels based on gellan gum with differently modified gelatin (ADH and CDH) that supports 3D cell culturing. The reporter line was also utilized as a stable imaging platform to monitor the cytotoxicity of CMs treated with cardiotoxic drugs such as doxorubicin, sunitinib, sorafenib, amiodarone, and astemizole. The reporter fluorescence enabled the direct visualization of the sarcomere organization and captured the myofilament deterioration and diminishing cross-striations in real time. The fluorescence intensity plot during the drug treatment can also be utilized as a direct measure of CM cytotoxicity. Our reporter CMs are an efficient in vitro model system for studying cardiac cell biology and drug toxicity.

## Materials and methods

### Generation of the MYH6-mScarlet hiPSC reporter line

A well-characterized, normal karyotype, integration-free hiPSC cell line UTA.04602.WT (Lahti et al. 2012; Ojala et al. 2016) generated from skin fibroblasts using pMX retroviral vectors OCT4, KLF4, c-MYC, and SOX2 as described earlier (Takahashi and Yamanaka 2006) was used for the generation of reporter clone. A detailed description of hiPSC line culture and cardiac differentiation is provided in the supplementary information. The CRISPR knock-in strategy for the generation of reporter line was designed on a plasmid-based system employing hCas9 (Addgene #41,815) (Mali et al. 2013). The construction of CRISPR-Cas9 vectors is provided in detail in the supplementary information. Gene editing of UTA.04602.WT was performed as previously described with slight modifications (Byrne et al. 2014). Briefly, the human iPS cells expanded on Geltrex in feeder-conditioned medium were pretreated with 10 μM ROCK inhibitor (Tocris Bioscience, Bristol, UK) in mTeSR1 before nucleofection. Cells were digested with versene to form single cell suspension followed by centrifugation for 3 min at 110 × g. After harvesting, 0.5 × 10^6^ cells were nucleofected (Amaxa 4D-Nucleofector X Unit, program CB-150) using P3 Primary Cell 4D-Nucleofection Kit (V4XP-3032, Lonza, Basel, Switzerland). Each nucleofection reaction contained 0.5 μg of hCas9, 1.5 μg of sgRNA-expressing plasmid, and 2 μg of target vector. Four to 5 days post nucleofection, selection was done by adding 0.5 μg/mL puromycin and the emerging antibiotic-resistant stem cell clones were manually picked and expanded. The genomic DNA was isolated by lysing the cells with DirectPCR Lysis Reagent Cell (PeqLab, Erlangen, Germany) supplemented with 0.2 μg/mL proteinase K (Roche, Mannheim, Germany) following the manufacturer’s protocol. The reporter integration was screened using a PCR strategy designed to identify the in-frame positioning of the reporter cassette and the homozygous reporter clones. The following forward and reverse primers were used:

- Target specific PCR
- Target forward- CATCTGGTATGCTCAGAGCTGTCAGGT
- Target reverse- CATGTGCACCTTGAACCGCATGAACTCC
- PCR for homozygous clones
- WT forward- CTTCGAGCCAAGAGCCGTGACATTGGTG
- WT reverse- TGGAGAGTGGCTTCAACTTCGGCACCA

The homozygous clones were further validated by DNA sequencing to exclude the presence of indels at the recombination site. The clone D103 was selected and the puromycin selection cassette in the hiPSC line was removed by transient expression of a Cre plasmid (1 μg, pCAG-Cre-IRES2-GFP Addgene # 26,646) as described above. The Cre removal of the selection cassette in the colonies was validated by PCR screening. The clone D103-4 was chosen, and its karyotype was analyzed using the hPSC Genetic Analysis Kit (STEMCELL Technologies). The CRISPR off-target side effects were checked by sequencing the 3 most likely off-targets and 2 additional predicted exonic off-targets. All predicted sites were free of mutations. The pluripotency of the genome-edited cardiac reporter line D103-4 was verified by PCR and immunocytochemistry as described before (Ojala et al. 2012). The details are provided in the supplementary information.

### Time-lapse live cell imaging

The Nikon Biostation CT automated imaging and analysis system was used for monitoring the reporter expression in the CMs during the cardiac differentiation course, biomaterial testing, and drug toxicity assay. The cells were imaged in culture over time and were maintained at 37 °C with 5% CO2 and 85% humidity.

### Immunocytochemistry and flow cytometry

Dissociated CMs were fixed with 4% PFA and stained with troponin T (cTnT, 1:2000, ab64623, Abcam, Cambridge, USA), MYBPC (1:400, sc-166081, Santa Cruz Biotechnology, TX, USA), connexin 43 (Cx43, 1:1000, C6219, Merk), and MYH6 (1:500, R&D Systems, Minneapolis, USA) primary antibodies, followed by labeling with secondary antibodies: Alexa Fluor 488–conjugated anti-goat, anti-mouse, and anti-rabbit antibodies respectively (1:800, A11055, A21202, A21206, Thermo Fisher Scientific). Images were obtained with Nikon N-SIM (super-resolution system microscope equipped with EM CCD camera iXon3 DU-897E (Andor Technology Ltd, Belfast, UK)). In-house-built selective plane illumination microscopy (SPIM) fluorescent imaging system was used to create the 3D images of the reporter CM cluster (Vuornos et al. 2019).

Cells at day 15 of CM differentiation were fixed in 4% paraformaldehyde and permeabilized by FACS buffer with 0.1% Triton™ X-100. Troponin T antibody diluted in FACS buffer (1:100) as primary antibody and Alexa Fluor 647–conjugated anti-goat (1:800, Thermo Fisher Scientific) as secondary antibody were used for FACS analysis. The resulting data were analyzed using FlowJo v8.5.2.

### Patch clamp recording and calcium imaging

The action potentials (APs) were recorded in perforated patch configuration using Amphotericin B in gap-free mode from spontaneously beating hiPSC-CMs. Data acquisition was conducted using Axon Series 200B patch-clamp amplifier connected to Digidata 1440a AD/DA converter driven by pCLAMP 10.2 software (Molecular Devices, San Jose, CA, USA). Calcium imaging was done using 4 μM Fluo-4 AM (Life Technologies Ltd). Detailed protocol for patch clamping and calcium imaging is provided in the supplementary information.

### Biomaterials and drugs used in the study

The five different biomaterials used in this study to investigate the maturation and migration of CMs are gelatin coating (Sigma-Aldrich); Biolaminin 521 LN coating (Biolamina, Sundbyberg Sweden), polyethylene terephthalate (PET)–based textile generated at Tampere University, Finland (Pekkanen-Mattila et al. 2019); and hydrogels CDH (60 mg/mL carbodihydrazide modified gelatin and 40 mg/mL oxidized gellan gum) and ADH (40 mg/mL adipic dihydrazide modified gelatin and 20 mg/mL oxidized gellan gum) produced at Tampere University (Koivisto et al. 2019). These gelatin–gellan gums are later referred to as CDH and ADH, respectively.

Doxorubicin, sunitinib, sorafenib, amiodarone, and astemizole that were used for the cardiotoxicity assay were purchased from Merk Life Science, NJ, USA.

### Magnetic-activated cell sorting and cell seeding

The CMs on day 30 of small molecule differentiation (SM-D) were dissociated into single cells using Multi Tissue Dissection Kit 3 (Miltenyi Biotec, Bergisch Gladbach, Germany) following the manufacturer’s instructions and were separated from other cell types using PSC-Derived Cardiomyocyte Isolation Kit, human (Miltenyi Biotec) and MACS buffer following the manufacturer’s instructions by magnetic-activated cell sorting (MACS). After cell sorting, the cells were resuspended in the suspension medium containing KnockOut DMEM with 10% fetal bovine serum (Biosera, Nuaille, France), 1% non-essential amino acids (NEAA), 1% GlutaMAX-I (100 ×) (all from Thermo Fisher Scientific, Gibco, Billings, Montana, USA), and 0.5% penicillin/streptomycin (Lonza, Basel, Switzerland).

The sorted CMs were seeded on the precoated flat bottom Greiner CELLSTAR 48-multiwell plate (Sigma-Aldrich) at the density of 2.5 × 10^5^ cells/cm^2^ for cytocompatibility and functional analysis. Precoating was done with 0.1% gelatin and Biolaminin 521 LN (15 μg/mL in dPBS). To increase the cell attachment, PET scaffold was coated prior with a thin layer of 0.1% gelatin without compromising the textile topography. For the hydrogel cytocompatibility analysis, single cell sorted CMs and beating CM aggregates (cut and isolated from the differentiating cell plate) were encapsulated in the CDH and ADH gel by mixing 30 μL of cell suspension in 150 μL GELA-ADH or GELA-CDH quickly followed by 150 μL oxidized gellan gum to form a total of 330 μL of hydrogel. Cell culture medium was applied on top of the samples after ∼20 min of gelation time. Unmodified gelatin coating was used as a negative control for the hydrogel CM experiment.

### RT-PCR and quantitative PCR

Total RNA from the CMs cultured in different biomaterials for 20 days was isolated using the Qiagen RNeasy Kit (Qiagen). For the RNA extraction of CMs in the hydrogel, the medium was removed, and the hydrogel was washed in dPBS three times. The CM aggregates were cut from the hydrogel with a scalpel under a microscope and collected in a microcentrifuge tube. In the case of single cell CM encapsulated in hydrogel, the entire hydrogel was used for the extraction. In order to increase the yield of extracted RNA, the hydrogel surrounding the cluster and single cells was partially digested by adding 100 μl of pronase solution (10 mg/mL stock solution prepared in DNase and RNase-free water, Merck) and placing it in a 37 °C water bath for 5 min with gentle agitation in between. The hydrogel solution was then added directly to the RNeasy® lysis buffer and homogenized and RNA was extracted according to the manufacturer’s instructions. The cells cultured in other biomaterials were lysed in RNeasy® lysis buffer following the protocol. DNase I–treated total RNA was then reverse transcribed using a High-Capacity cDNA Reverse Transcription Kit (Applied Biosystems, CA, USA) according to the manufacturer’s instructions.

cDNA was amplified by the TaqMan Universal Master Mix (Applied Biosystems) using the BioRad CFX384 Real-Time PCR Detection System. TATA-box binding protein (TBP), eukaryotic translation elongation factor 1 alpha1 (EEF1A1), and glyceraldehyde-3-phosphate dehydrogenase (GAPDH) were used as endogenous control genes for data normalization. All samples were analyzed in triplicates and the relative quantification was calculated by the ∆∆CT method (Livak and Schmittgen 2001). MACS-sorted hiPSC-CMs at day 30 of differentiation was used as the calibrator sample to assess the relative fold expression. Cells were collected from three biological replicates and the fold change is expressed as mean ± standard error of the mean (SEM). TaqMan assays used in the qPCR protocol are presented in Table S6.

### Statistical analysis

The statistical significance of the differences in electrophysiological parameters between the WT and D103-4 CMs was analyzed by unpaired two-tailed *t* test. For gene expression analysis, the Kruskal–Wallis test followed by Dunn’s test correction for multiple comparisons was used where *p* < 0.05 was considered statistically significant. Statistical analysis was conducted in Prism 5 for Windows v5.02 (GraphPad Software, Inc.).

### Drug treatment and cell viability assay

The drug stocks, prepared in DMSO or dH2O at10 mM, were equilibrated to 37°C and diluted in EB medium to 2× target concentration. The reporter hiPSC-CMs cultured in both 2D and 3D were exposed to the respective drugs by diluting 1:1 with the media for 120 h. DMSO was used as the vehicle negative control in the drug toxicity assays. For the cell viability assay, hiPSC-CMs at day 30 of differentiation were plated on Geltrex coated 384-well plate at 25,000 cells/well. Cells were treated with increasing doses of drug concentration between 0 and 100 μM for 120 h. ATP present in metabolically active cells was quantified using the CellTiter-Glo Luminescent Cell Viability Assay (Promega, WI, USA) according to the manufacturer’s instructions. Luminescence was quantified using Victor^2^14202 Multilabel counter (Wallac, PerkinElmer) and was normalized to the values of the vehicle control. The mScarlet fluorescence intensity of the CMs was measured from the fluorescence microscopy images at 0 h and after 120 h of drug treatment using the Fiji Image processing software (Schindelin et al. 2012). The corrected total cell fluorescence for each region of interest at 120 h of the respective drug dose was quantified and normalized to the values obtained for the same at 0 h. Data for each drug dose (*n* = 30, from three different biological replicates) was plotted and is presented as mean ± standard deviation (SD).

## Results

### Generation of MYH6-mScarlet cardiac iPSC reporter line

**Fig. 1:**
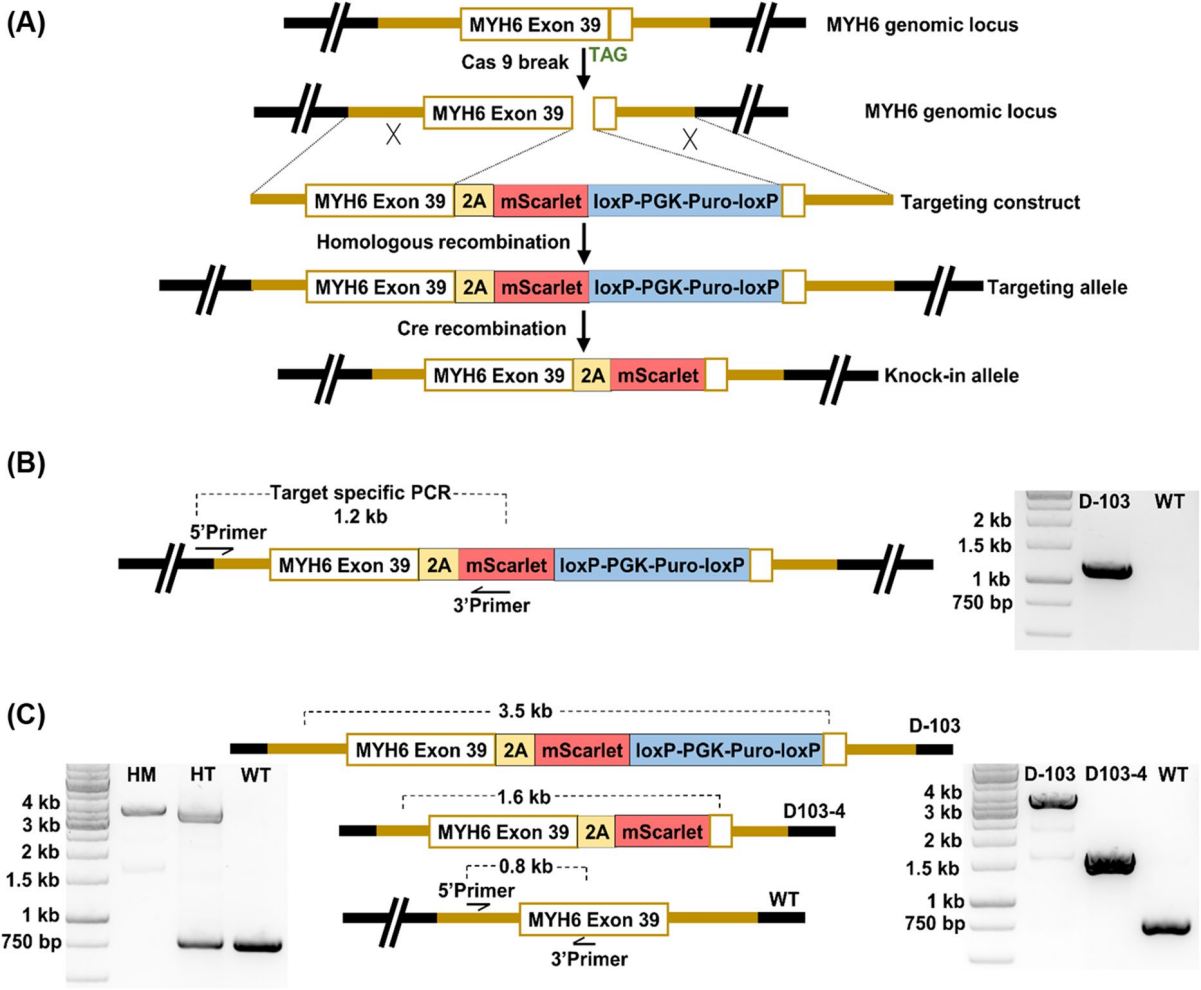
Generation of MYH6-mScarlet iPSC reporter line. **A** Schematic illustration depicting the MYH6-mscarlet reporter design. **B** The target-specific PCR designed for screening the reporter integration that spans outside the 5′ homology arm up to the 2A-mScarlet region gives an amplicon of 1.2 kb. The parallel gel image shows the positive amplification of target PCR in the reporter clone D-103. **C** PCR strategy to identify the homozygosity and Cre removal of puromycin selection cassette. The primers spanning the integration region were used to amplify the genomic DNA from the knock-in clones. Homozygous clone (HM) with the modified locus in both alleles produces only one amplicon of 3.5 kb that comprises the knock-in cassette, whereas the heterozygous clone (HT) produces two amplicons from the modified and wild-type (WT) parental locus of 3.5 kb and 0.8 kb respectively (left panel gel image). The removal of puromycin selection cassette reduces the amplicon length of the modified locus to 1.6 kb as shown in the right panel gel image

Using CRISPR-Cas9 genome editing technology, we targeted the myosin heavy chain 6 (*MYH6*) gene locus of the hiPSC cell line UTA.04602.WT (Lahti et al. 2012), to generate a knock-in mScarlet CM reporter line. MYH6 that encodes the alpha heavy chain subunit of cardiac myosin is a highly specific cardiac marker whose expression begins early in CM differentiation and continues in adult cells (Franco et al. 1998). We chose the fluorescent tag mScarlet for constructing our reporter line due to its favorable spectroscopic properties particularly the brightness and photostability that could benefit the generation of trackable CMs. The reporter knock-in strategy used in this study is shown in Fig. 1A. The positive hiPSC clones were screened for reporter integration by a target PCR designed to span outside the 5′ homology arm up to the 2A-mScarlet region (1.2 kb) as shown in Fig. 1B. Over 95% of the screened clones were positive for the correct in-frame knock-in of the reporter cassette confirming high gene targeting efficiency. To obtain brighter mScarlet reporter activity, homozygous knock-in clones were identified by PCR targeting the wild-type MYH6 gene locus (Fig. 1C). The homozygous clone gave an amplicon size of 3.5 kb and lacked the wild-type MYH6 band (0.8 kb), whereas the heterozygous clone amplified both amplicons as shown in the gel image of Fig. 1C. The homozygous clone named D-103, free of predicted off-target mutations and indels, was selected and the puromycin selection cassette was successfully removed by Cre recombinase. The excision of the antibiotic selection cassette was confirmed by the reduced wild-type PCR amplicon of 1.6 kb (Fig. 1C). The final reporter clone D103-4 was selected for all the subsequent cardiac differentiation experiments.

The hiPSC reporter clone D103-4 was characterized for the characteristic markers of pluripotency at the protein (Fig. S1A) and gene (Fig. S1B) level to confirm that the stem cell property has not been altered by gene editing and subsequent antibiotic selection. It was shown that the cell line formed colonies expressing Nanog, OCT4, SOX2, TRA-1–60, and TRA-1–81 by immunofluorescence and expressed endogenous *Nanog*, *OCT4*, *REX1*, *SOX2*, and *c-MYC* as confirmed by RT-PCR. The genomic integrity of the reporter line was assessed as the cell line is at risk of developing aneuploidy when maintained in culture for many passages for the genome editing process. We analyzed the majority of hiPSC karyotypic abnormalities reported so far in our reporter line and showed that D103-4 maintains a normal karyotype (Fig. S1C).

## Cardiomyocyte differentiation validates the mScarlet reporter activity in D013-4

**Fig. 2:**
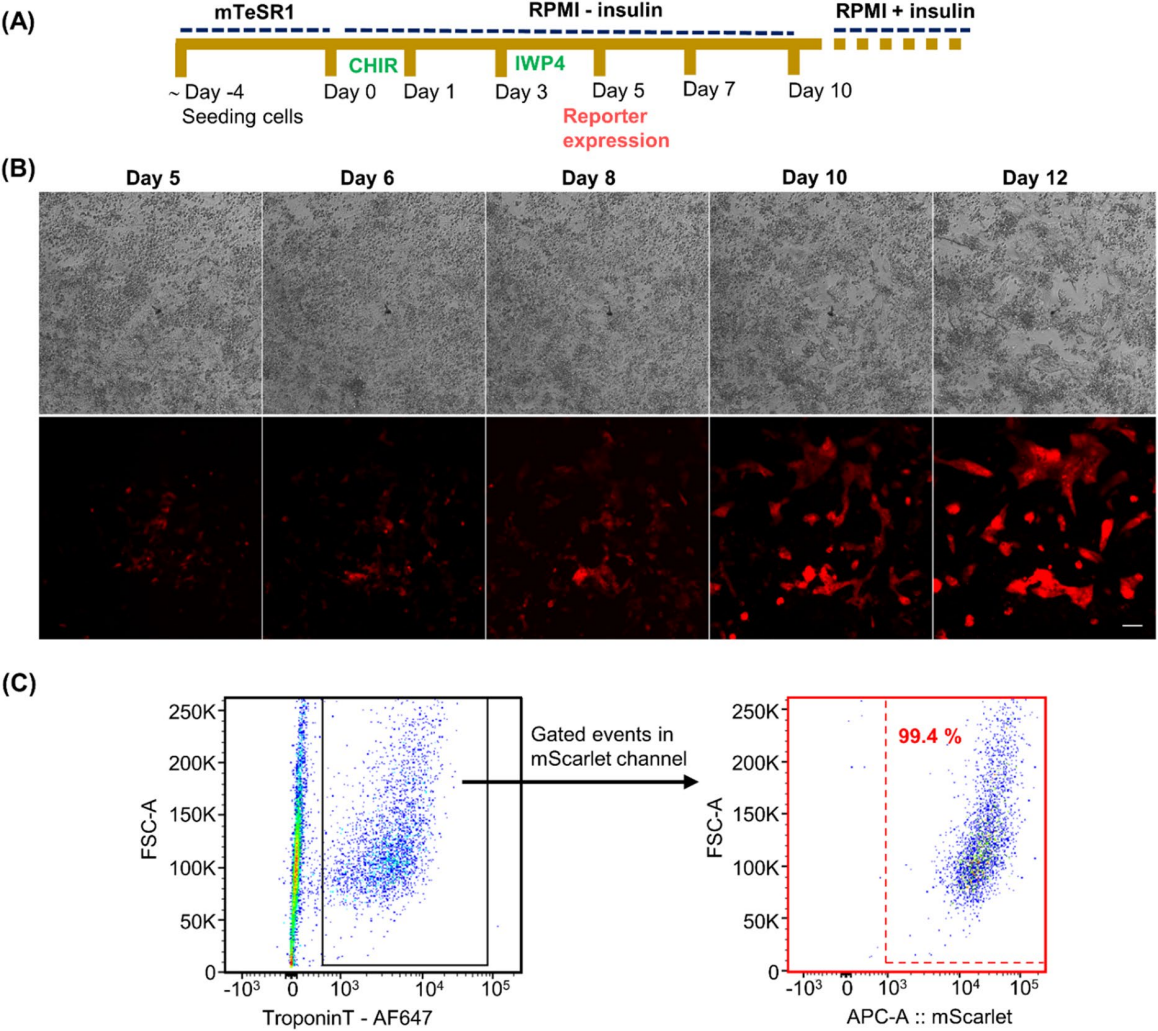
Cardiac differentiation of MYH6-mScarlet cardiac iPSC reporter line confirming the reporter activity. **A** Schematic representation of the cardiac small molecule differentiation protocol used in the study. **B** MYH6-mScarlet reporter expression profile monitored during CM differentiation course by a time-lapse imaging system; scale bar is 100 μm. **C** Flow cytometry analysis of the reporter D103-4 hiPSC line at day 15 of differentiation showing the percentage of troponin-positive CMs that are mScarlet-positive confirming the cardiac-specific expression of the reporter. Troponin-positive cells were gated from the mixed population and were analyzed for the expression of mScarlet reporter. **C**HIR, glycogen synthase kinase 3β inhibitor-CHIR 9902; IWP4, inhibitor of WNT production-4

To test the cardiac reporter activity in CRISPR-Cas9-generated D103-4, CM differentiation was initiated by small molecule differentiation (SM-D) that functions by the temporal modulation of regulators of canonical Wnt signaling (Lian et al. 2012) (Fig. 2A). We used high-precision time-lapse microscopy to characterize and track CMs during the differentiation of hiPSCs towards the cardiac lineage (Fig. 2B). The mScarlet reporter expression could faithfully recapitulate endogenous activation of the target gene as initial mScarlet-positive cells were evident from day 5 and more positive cells appeared at later time points with maximal expression on day 14. Time-lapse images from day 1 to day 4 of CM differentiation are shown in Fig. S2. The progression of the reporter activity in D103-4 cells was visualized in real time, providing an optimal fluorescent tracer specific for CM live imaging. The fluorescent signal labeled the entire cell body of the CM and mScarlet-positive cells tended to cluster together initiating the CMs beating activity. The first beating cluster of cells was observed on day 8, and by day 12, robust spontaneous contractions were visible. The cardiac reporter activity in D103-4 hiPSC line was also confirmed by using multiple differentiation methods (Fig. S3). From the flow cytometry analysis, almost 99.4% of cardiac troponin T (cTnT)–positive cells were mScarlet-positive, confirming the cardiac-specific expression of mScarlet (Fig. 2C).

### Structural and electrophysiological characterization of reporter CMs

**Fig. 3:**
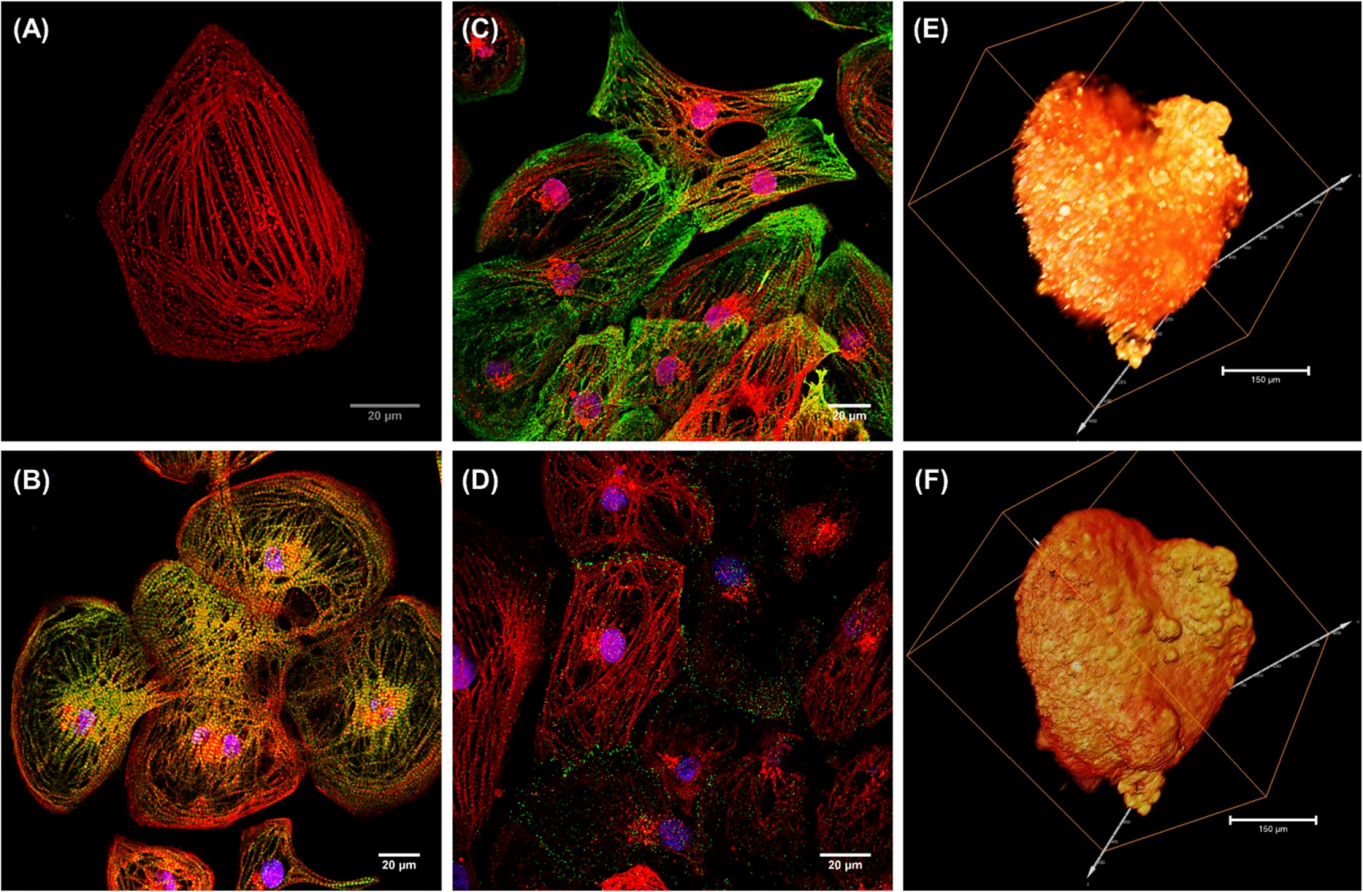
Immunofluorescence images of MYH6-mScarlet reporter CMs expressing cardiac structural markers. Confocal image of fixed reporter CMs showing the myofibril striations (**A**). Reporter CMs immunostained with cardiac troponin T (cTnT) green (**B)**, myosin-binding protein (MYBPC) green (**C)**, and gap junction connexin 43 (Cx43) green (**D)**. Nuclei were counterstained with DAPI (blue) in all immunostained images. Scale bars are 20 μm. Single/selective plane illumination microscopy (SPIM) optical 3D stack of multi-focal mScarlet fluorescence showing the spatial distribution (**E)** and topography of cardiomyocytes in an aggregate within cluster (**F)**

The MYH6-mScarlet reporter CMs exhibit cardiac structural markers and recapitulates the electrophysiological properties of the parent hiPSC line. The knocked-in mScarlet fluorescence tag enabled the direct visualization of the myocardial architecture of CMs without staining (Fig. 3A). Even though the 2A self-cleaving peptide was incorporated between the proteins to prevent interference with the proper MYH6 function, the cleavage efficiency was not 100% in our construct. The mScarlet CMs displayed organized sarcomeric structures in the confocal microscopy and immunostaining with anti-MYH6 demonstrates significant areas of colocalization suggesting that majority of the mScarlet exist as MYH6-mscarlet fusion protein (Fig. S4). The differentiated CMs were characterized for the expression of myofilament proteins by immunofluorescence staining of troponin T (cTnT), myosin-binding protein C (MYBPC), and ventricular gap junction protein connexin 43 (Cx43) responsible for electrical coupling (Fig. 3B, C, D respectively). Single/selective plane illumination microscopy (SPIM) optical 3D imaging technique was used to visualize the distribution, density, and topography of CM clusters in 3D culture (Fig. 3E, F). The mScarlet fluorescence permitted the macroscopic visualization of the 3D structural organization of CMs within a cluster (Movie 1).

**Fig. 4:**
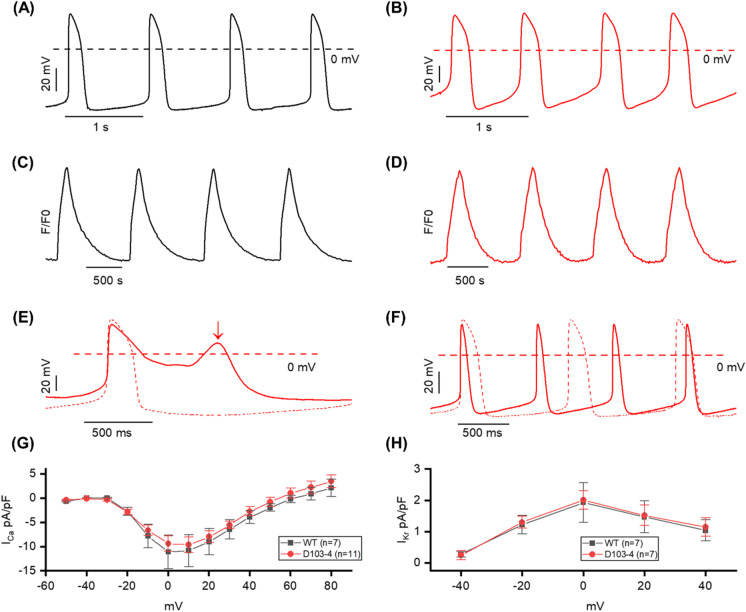
Electrophysiological characteristics and comparisons of D103-4 with WT. **A, B** Spontaneous action potential (AP) recording from WT (black) and D103-4 (red) CMs. **C**, **D** Intracellular calcium dynamics recording from WT (black) and D103-4 (red) CMs. **E** Application of 1 μM E-4031 drug induced the early after depolarization (EAD) in D103-4. Dashed line and solid line represent the before and after application of 1 μM E-4031. Arrow indicates the EAD. **F** Application of 100 nM adrenaline in spontaneous beating. Dashed line and solid line represent the before and after application of adrenaline. Note that the beat rate was increased, and AP duration was shortened after the application of adrenaline. **G** Current–voltage relationship of calcium current densities (ICa) recorded from WT (black) and D103-4 (red). **H** Current–voltage relationship of peak current densities of rapid delayed rectifying potassium current (IKr) recorded from WT (black) and D103-4 (red)

Action potentials (APs) were recorded from spontaneously beating WT and D103-4 CMs and the representative traces of AP from WT (black) and D103-4 (red) are shown in Fig. 4A and B. To verify whether the mScarlet knock-in at the MYH6 genome locus altered the AP behavior, various AP parameters were compared between WT and D103-4 CMs. Only ventricular-like hiPSC-CMs characterized by APD90/APD50 < 1.3 and APA > 90 mV were used for the comparison. Statistical analysis did not show any significant difference in AP parameters between WT and D103-4 (ns, *t* test) (Table S1). In addition, intracellular calcium (Ca2 +) dynamics were also measured using fluo-4 from both WT and D103-4 and the representative traces of Ca2 + transients from WT and D103-4, displaying similar Ca2 + handling as shown in Fig. 4C and D. The mScarlet fluorescence did not interfere with the fluo-4 loading and Ca2 + measurements in the reporter CMs (Table S2). We also examined the effect of 1 μM E-4031 (rapidly activating delayed rectifier potassium (K +) current (IKr) blocker) on spontaneously beating D103-4 CMs, which induced an early after depolarization (EAD) that confirmed the reporter CMs response to pharmacological testing (Fig. 4E). Furthermore, we also examined the effect of 100 nM adrenaline in spontaneously beating and stimulated D103-4 CMs. Adrenaline caused an increase in the beat rate in spontaneously beating D103-4 CMs and shortening of action potential duration (APD) in both spontaneously beating and stimulated D103-4 CMs exhibiting the response of reporter CMs to the adrenergic stimulation (Fig. 4F). In addition, we also measured the ionic currents from WT and D103-4 CMs. Figure 4G depicts the current–voltage relationship of Ca2 + current densities (ICa) from WT and D103-4 CMs which showed no statistical differences in ICa between the CMs at any tested potentials. IKr from WT and D103-4 CMs were also measured (Fig. 4H) and our results showed no statistical differences in peak and tail IKr at any tested potential (Table S3) which confirms that the CRISPR genome editing did not alter the electrophysiological properties of the differentiated CMs.

### Reporter CMs as a tracking tool in biomaterial scaffolds

**Fig. 5:**
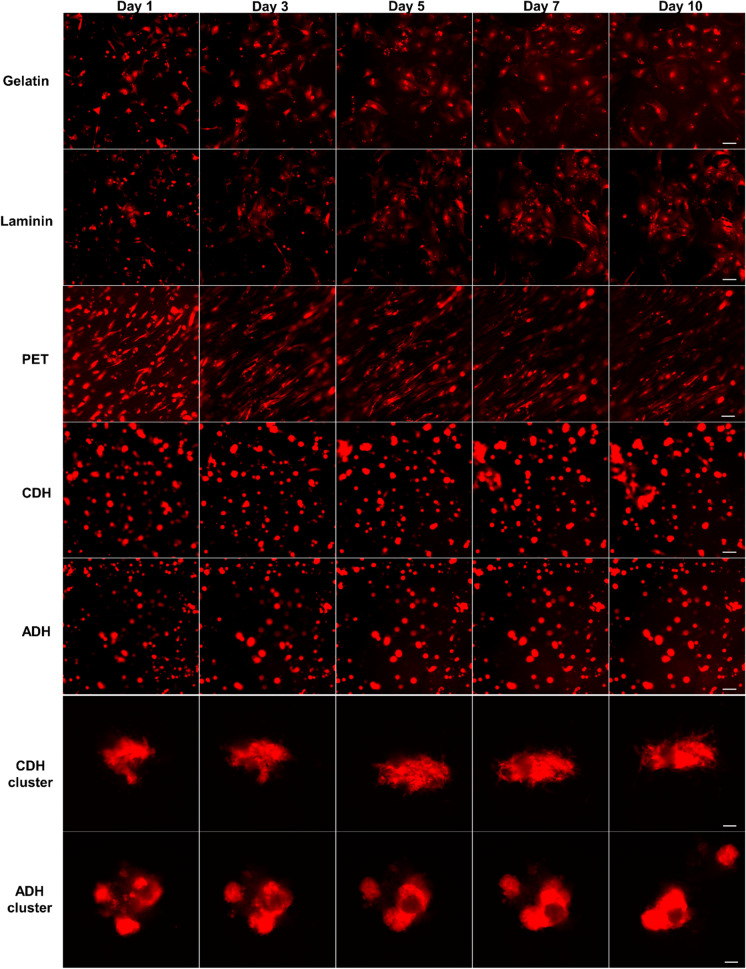
Tracking CMs location, migration, and behavior on different biomaterial scaffolds using time-lapse live cell imaging. HiPSC-CMs sorted at day 30 of differentiation were seeded into gelatin, laminin, PET, ADH, and CDH hydrogel and were tracked for 10 days. The mScarlet reporter expression was monitored for visualizing the morphology and attachment of hiPSC-CMs in 2D and 3D culture systems. Scale bar is 100 μm

The architecture of a biomaterial provides topographical cues that can influence the overall alignment of CMs. This can impact the contractile strength and electromechanical coupling of CMs, promoting the maturation state of hiPSC-CMs. In this study, we used our reporter CMs to monitor the morphology and alignment of differentiated CMs in five different biomaterials including gelatin, laminin, PET, and ADH and CDH gelatin–gellan gum hydrogels for 10 days by live cell imaging (Fig. 5). In the 2D culture system (gelatin and laminin), the CMs displayed great cell attachment and started to spread from day 1 of seeding. The cells attained a polygonal morphology, showing a vast area of attachment to the substrate, and beating was observed from day 2. In both the 2D substrates, cells change their phenotype acquiring random shapes and orientations, formed pseudopods, and were flattened as the culture continued. However, compared to laminin, CMs in gelatin substrate were more scattered and exhibited a rather flat and round shape. The reporter CMs cultured on the PET textile could be clearly visualized and the background blue color of the material did not hinder the imaging. CMs were elongated along the textile fibers from day 1 with better cell alignment compared to cells on the 2D scaffolds (Fig. 5, PET panel). After seeding, only subtle contractions were observed on day 2 and the number of beating cells continued to increase over the following 4–5 days. The topography of the textile characterized by fiber size and weaving patterns facilitated the alignment and elongation of hiPSC-CMs with good beating functions, exhibiting more mature morphology. For the 3D culture on the gelatin–gellan gum hydrogels, both MACS-sorted single cells and beating CM aggregates that were directly cut and isolated from the differentiation plate were seeded. The mScarlet reporter CMs encapsulated within the hydrogel could be easily tracked, and the heterogeneous nature of the hydrogel did not limit its fluorescence imaging in real time. In the case of single CMs, most of the cells appeared rounded and only few were elongated. Subtle beating of single CMs was observed in the 3D hydrogel, and over the days, cells migrated through the hydrogel to form small clusters with robust beating. Compared to ADH, CDH hydrogel accelerated these cells clustering in the single seeded CMs (Fig. 5, CDH and ADH panels). CM aggregates seeded in the hydrogel recovered their beating phenotype after an overnight culture and both the hydrogels maintained the spontaneous contractions of encapsulated CMs throughout the culture and supported their adhesion. The applicability of the reporter CMs in probing the effect of biomaterials on cell behavior allows us to better characterize various scaffolds for CM structure and morphology.

### Influence of biomaterials on the maturation status of hiPSC-CMs

**Fig. 6:**
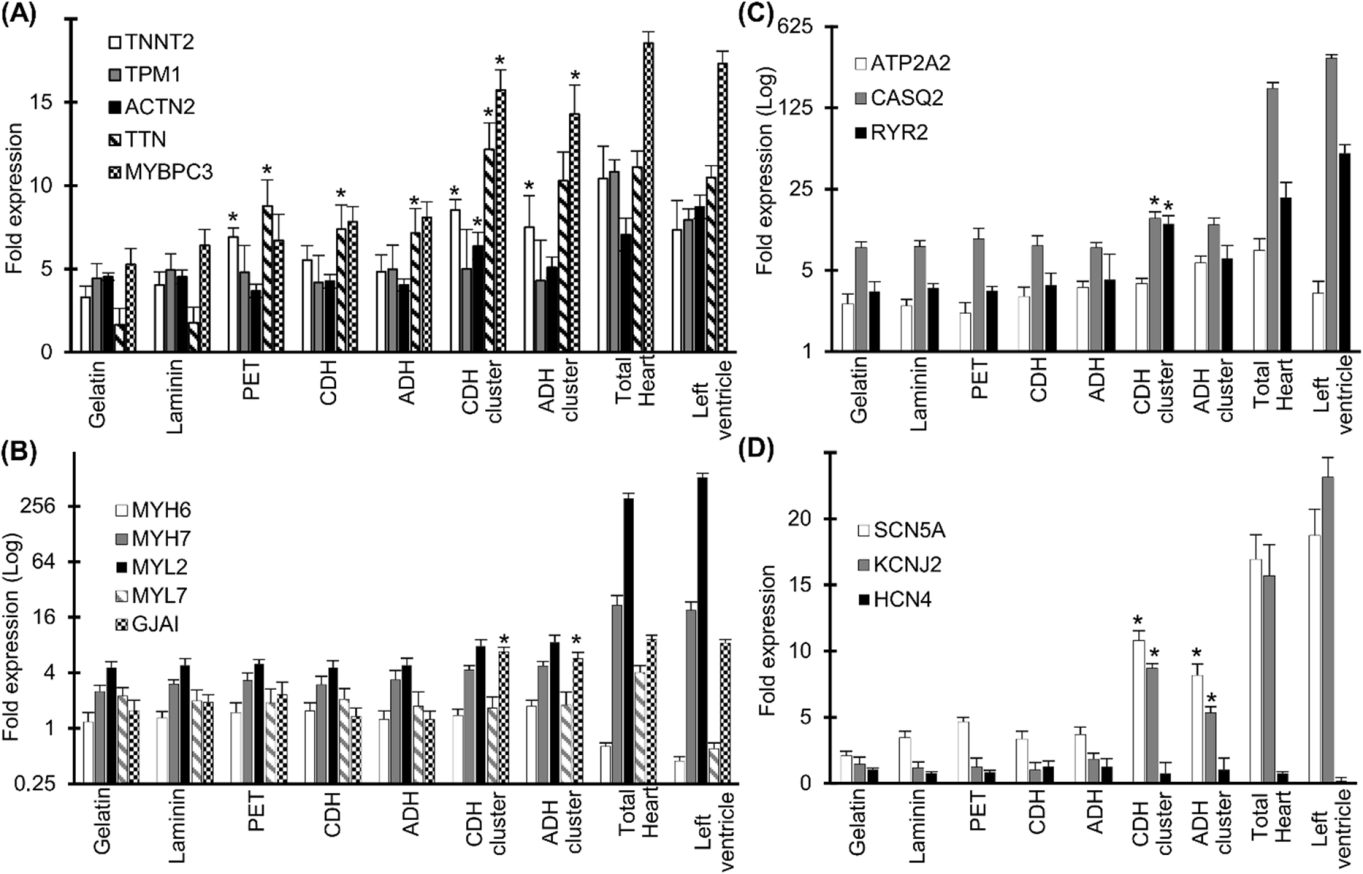
3D cultured hiPSC-CMs upregulate the key cardiac structural and functional genes. Quantitative reverse-transcription polymerase chain reaction (qRT-PCR) data for a panel of key cardiac markers: major troponins and myosin-related genes (**A**, **B)** and major ion channels expressed in hiPSC-CMs cultured on gelatin, laminin, PET, and ADH/CDH gelatin–gellan gum hydrogels (**C**, **D)**. The expression of the same genes in adult total heart CMs and left ventricle are shown in parallel. The qRT-PCR data are presented as the fold change relative to the MACS-sorted hiPSC-CMs at day 30 of differentiation (before seeding to the biomaterials). CM clusters cultured on the 3D gelatin–gellan gum hydrogels showed a significant increase in the expression levels of cardiac type troponin T2 (*TNNT2*), myosin-binding protein C (*MYBPC*), titin (TTN), and cardiac gap junction protein Cx43 (GJAI), when compared to the CM 2D monolayers (gelatin and laminin). Differences in the gene expression profile between CMs cultured in 2D and 3D culture systems were analyzed by Kruskal–Wallis test and post-hoc Dunn’s test (*p < 0.05). Data expressed as mean ± SEM, *n* = 3 biological replicates.

To determine the effect of culturing in different biomaterial scaffolds on the maturation status of hiPSC-CMs, we used qRT-PCR to quantify the relative expression of certain key cardiac structural and functional genes after 20 days in culture. As a benchmark, the expression of the same markers in adult total heart CMs and the left ventricle was measured. We assayed genes related to sarcomeres, Ca2 + handling, sodium (Na +), and K + ion channels. The cardiac gene expression profile showed no significant difference between gelatin and laminin-plated CMs. However, compared to the 2D culture environment, the gene expression analysis showed a substantial increase in the expression of *TNNT2*, myosin-binding protein C (*MYBPC*), *ACTN2*, and titin (*TTN*) in the 3D hydrogel cultured CM aggregates (Fig. 6A). Within the 3D hydrogels, the cardiac gene expression profile of the CM aggregates was more robust compared to the single cell CMs. We observed significant induction in the cardiac gap junction protein Cx43 (*GJAI*) expression in the CM clusters suggesting functional electromechanical coupling between cells in a 3D culture system (Fig. 6B). Expression of the Ca2 + handling and ion channel genes like *RYR2*, *SCN5A*, *KCNJ2*, and *CASQ2* was also upregulated in CM clusters encapsulated in the CDH hydrogel (Fig. 6C and D). The qRT-PCR data suggest that the 3D cardio-biomimetic microenvironment provided by the gelatin–gellan gum hydrogel can greatly enhance the maturation of hiPSC-CMs.

### Reporter CMs as an imaging platform to assess drug-induced structural cardiotoxicity

**Fig. 7:**
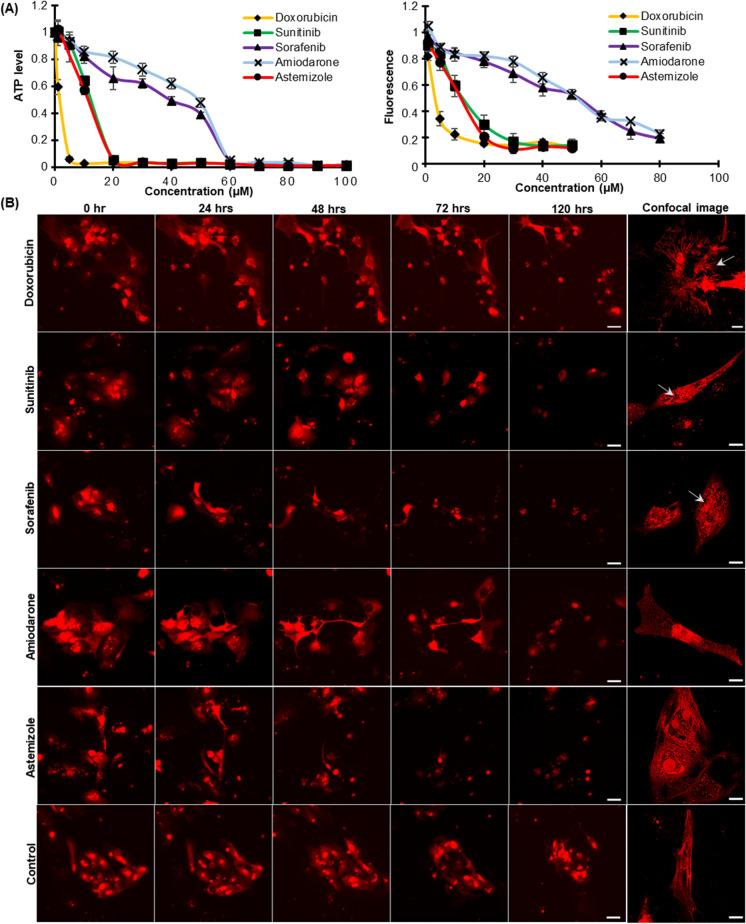
Monitoring drug-induced structural cardiotoxicity using the reporter CMs. **A** Dose response curves of doxorubicin, sunitinib, sorafenib, amiodarone, and astemizole after 120 h of drug treatment (0 to 100 μM) in the reporter CMs using CellTiter-Glo® luminescence–based cell viability assay (left panel) and by measuring the mScarlet fluorescent intensity (right panel). The ATP level of each drug dose was normalized to the vehicle control, and for the fluorescence plot, each drug dose value was normalized to the values obtained for the same at 0 h of drug treatment. Data are presented as mean ± SD. **B** Time-lapse imaging of the progression of cytotoxicity in the reporter CMs treated with drug dose that depletes 100% ATP level in 120 h. Scale bar is 100 μm. Confocal images of the reporter CMs showing specific structural cardiotoxicity after 72 h of respective drug treatment are shown in the right-most panel of each row. Scale bar is 20 μm

Preservation of cell morphology is important for the basal function of the cardiomyocyte. Hence, an efficient assessment of cardiotoxicity during the drug development process should account for the structural toxicity along with the functional assays as myofibril loss and sarcomere disarray are the most obvious phenotypic changes in human cardiomyopathy and failing hearts. To this end, we utilized our reporter CMs as a real-time cardiac imaging system to track drug-induced perturbations in CM morphology in both 2D and 3D cell culture models (Fig. 7B and Fig. S4). We selected five drugs with known adverse cardiac side effects: doxorubicin, an anthracycline, typically associated with dose-dependent cardiomyopathy and congestive heart failure; sunitinib and sorafenib, small molecule multi-targeted kinase inhibitors (TKI) associated with significant incidence of cardiovascular dysfunction and reduced left ventricular ejection fraction; amiodarone, a class III anti-arrhythmic drug which has been reported to cause electrophysiological effects, such as bradycardia and heart block; astemizole, an anti-histamine which has been withdrawn from the market due to adverse cardiovascular events like cardiac arrest, QT prolongation, and Torsades de pointes. Changes in cellular ATP levels were measured as a function of drug dose and time using the CellTitre-Glo assay by exposing the reporter CMs to each drug (doses ranging between 1 and 100 μM) for 120 h (Fig. 7A, left panel). The assay resulted in a dose-dependent decrease in cell viability compared to the vehicle-treated control cells. The level of mScarlet cellular fluorescence in the CMs was also measured from the fluorescence microscopy images at 0 h and after 120 h of each drug treatment (Fig. 7A, right panel). The drug-treated reporter CMs exhibited a dose-dependent decrease in mScarlet fluorescence compared to the vehicle control CMs that did not show any significant difference in the fluorescence intensity after 120 h of treatment (Fig. S5). The fluorescence intensity plot parallels with the luminescence assay and hence can be utilized as a simpler method for the direct evaluation of CM cytotoxicity without sacrificing the cells. The shutdown of mScarlet fluorescence due to doxorubicin toxicity can be clearly visualized compared to the vehicle-treated control CMs in Movies 2 and 3. For each drug, the concentration that resulted in near 100% depletion of ATP in 120 h was selected for visualizing the changes in the myocardial structure that leads to apoptosis. Time-lapse imaging of the reporter CMs enabled tracking of the progression of toxicity with exposure time in the same population of CMs (Fig. 7B and Movies 4–9). Confocal microscopy after 72 h of drug treatment showed sarcomeric irregularities and myofibril deterioration in the drug-treated reporter CMs (Fig. 7B, respective right panel confocal images, and Fig. S6). As compared to the vehicle control CMs, doxorubicin-treated CMs demonstrated an elongated spindle-like cellular morphology with reduced cell size and significant reduction in cell–cell contacts. Sarcomeric disarray and myofilament damage could be clearly visualized in the drug-treated CMs (Fig. S6). Both the tyrosine kinase inhibitors sunitinib and sorafenib induced cytoplasmic vacuolation together with myofibril deterioration. Treatment of amiodarone and astemizole also induced the disintegration of myofilaments and sarcomere irregularities which are the most common structural changes in human cardiomyopathy. Drug-specific structural toxicity at the single cell level was distinguishable with the reporter CMs, which is crucial for the analysis of cardiotoxicity from different classes of drugs. Drug response of CMs in 3D culture environment was also monitored by capturing the mScarlet fluorescence (Fig. S7). While the vehicle control CM cluster had a compact, organized distribution of cells, the drug-treated cardiac clusters displayed a scattered distribution of necrotic cells with a significant loss of cell–cell contact. In both 2D and 3D culture environments, the mScarlet fluorescence enabled the visualization of cardiotoxic phenotypes in the myocardial structure without immunostaining, generating a faster and reliable imaging system to evaluate drug-induced structural cardiotoxicity.

## Discussion

Fluorescent imaging using our cardiac reporter line provides an unprecedented means to monitor the biology and function of hiPSC-CMs in real time. This stable imaging platform has huge potential in drug discovery studies as well as when analyzing cardiac regeneration or toxicity. Several groups have reported their success in constructing different reporter iPSC and ESC lines that express a variety of fluorescent markers (Den Hartogh et al. 2015; Garreta et al. 2016; Schwach et al. 2017; Tsai et al. 2020). We have generated an extremely bright hiPSC cardiac reporter line which is efficient for imaging CMs encapsulated in heterogeneous biomaterials using the monomeric red fluorescent protein tag mScarlet (Bindels et al. 2017) that has the highest recorded brightness, quantum yield, and fluorescent lifetime. Our reporter line can identify nascent CMs at day 5 of differentiation which is the fastest reporting time compared to other previously established cardiac reporters (Garreta et al. 2016; Tsai et al. 2020). The fast maturation time of mScarlet allowed the accurate measurement of rapid gene expression dynamics of MYH6 and effectively increased the fluorescence signal in differentiating cells. This confirms the ability of our reporter fluorescence to be used as a readout in screens for differentiation modulating compounds, optimizing efficient cardiac differentiation protocols, and isolating pure cardiac cell populations. It also produces clear and informative 3D images from CM clusters which can be utilized for imaging CMs in microfluidic devices and organ-on-chip platforms. Additionally, the fluorescence could display the myofibril structure in live CMs through confocal microscopy, broadening its application to sarcomere contractility studies. Compared to other cell tracking techniques, limitations like the half-life of the tracking label, dilution of fluorescence during cellular division, and the labeling of other non-specific cells after donor cell death are bypassed in this stable cardiac reporter line.

We studied the CMs’ behavior on different biomaterial scaffolds with these illuminated CMs and could observe differences in morphology, alignment, and migration of CMs. CMs encapsulated in 3D hydrogels clearly depicted enhanced mRNA levels of several key cardiac structural and functional genes compared to the traditional 2D culture. 3D hydrogel cultures especially CDH gelatin–gellan gum allowed CM extensions to become entangled within the extracellular matrix fibrils, resulting in mechanical interactions and anchorage that are not possible when cells attach to 2D planar surfaces. These hydrogels have been earlier proved to exhibit mechanical behavior, especially elasticity, which resembles cardiac tissue (Koivisto et al. 2019). Mechanical cues of biomaterials including rigidity, microstructure, and 3D architecture have shown to exert changes in intracellular cell signaling cascades and subsequently influence gene expression patterns (Jang et al. 2020; Naqvi and McNamara 2020; Wan et al. 2021). With these reporter CMs, it was also possible to investigate the guided behavior of hiPSC-CMs on the blue woven core-sheath fibers made of PET which is a non-transparent and difficult material to image cells due to its opaqueness and topographical structure (Pekkanen-Mattila et al. 2019). Fluorescent hiPSC-CMs were elongated and aligned along the long axis on the PET fibers indicating their structural maturation. While these different biomaterials could improve certain properties of hiPSC-CMs, none of them could generate CMs mature as native human adult CMs. Currently, multidisciplinary approach involving the combination of multiple cues in the cell microenvironment are widely being explored for hiPSC-CM maturation (Guo and Pu 2020; Karbassi et al. 2020). However, it is very challenging to monitor the dynamic changes of hiPSC-CMs, especially in co-culture systems and microfluidic organs-on-a-chip, where multiple cell interactions must be carefully studied (Garzoni et al. 2009; Iyer et al. 2009; Marsano et al. 2016; Suhaeri et al. 2017; Varzideh et al. 2019). Most of the cell behavior data from these studies lack information on the relationship between a phenotype and the cell location in the culture environment. Our reporter line can report cell fate in all culture systems, providing a vital tool for tracking the location, spatial distribution, and migration of CMs. It does not require fluorescent labeling and facilitates microscopic visualization without disrupting the scaffold structure.

We also demonstrated the potential of our reporter CMs as a quick and precise system for monitoring drug-induced cytotoxicity and cytoskeletal damage over an extended time. Following the treatment with different cardiotoxic drugs, we observed a dose-dependent effect on CM viability and changes in myocardial architecture. Over the past decade, many studies have shown the use of iPSC-CMs to assess the cardiac toxicity of different drugs (Narkar et al. 2022; Sharma et al. 2017; Thomas et al. 2021). However, monitoring live CMs at different drug concentrations gives an additional application of the reporter CMs on drug dose effects and identifies drugs that interfere with the CMs contractile machinery but may not directly impact the CMs electrophysiology and viability. One of the main advantages of reporter gene imaging is that only live CMs will produce the reporter protein, and this allows us to visualize CM apoptosis in real time. The phenotypic variations of the hiPSC-CMs in response to drugs will enable us to further probe the mechanism of distinct structural toxicities and identify specific signaling pathways associated with the toxicity. This imaging tool could also be further enhanced by combining it with other functional assays such as CM contractility, electrophysiology, and Ca2 + signaling which will provide complementary insights into the mechanism of drug-induced cardiotoxicity.

In conclusion, the study has generated a real-time 2D/3D cardiac imaging platform that enables non-invasive, continuous tracking and high-resolution imaging of cardiomyocytes, optimal for cytocompatibility analysis and drug toxicity screening. The cardiac-specific hiPSC reporter line that permits the visualization of CM morphology, distribution, and survival is a valuable multipurpose research tool in the cardiac iPSC field. Its highly efficient imaging capabilities in a 3D environment and the ability to provide real-time continuous monitoring of the cardiac stem cell fate can develop advanced bioengineered cardiac systems with improved CM maturation. Our reporter CMs will also be useful in transplantation studies as they offer real-time guidance on stem cell transplantation, cell transit, and engraftment in transplantation studies. Future work towards developing an organ-on-chip platform will further advance its application in drug development and precision medicine.

## Supplementary Information

Below is the link to the electronic supplementary material.

Supplementary file1 (DOCX 7.55 MB)

Supplementary file2 (MP4 4324 KB)

Supplementary file3 (MP4 1382 KB)

Supplementary file4 (MP4 2097 KB)

Supplementary file5 (MP4 929 KB)

Supplementary file6 (MP4 723 KB)

Supplementary file7 (MP4 567 KB)

Supplementary file8 (MP4 744 KB)

Supplementary file9 (MP4 592 KB)

Supplementary file10 (MP4 724 KB)

## Data Availability

The data used to support the findings of this study are available from the corresponding author upon request.
