# Supplementary material for: Fluorescent hiPSC-derived MYH6-mScarlet cardiomyocytes for real-time tracking, imaging, and cardiotoxicity assays
Source: Cell Biol Toxicol. 2022 Jul 23;39(1):145–63. doi: 10.1007/s10565-022-09742-0 (PMC10042918; doi:10.1007/s10565-022-09742-0)
Supplement: Supplementary file 1 — Supplementary file1 (DOCX 7.55 MB) [file 10565_2022_9742_MOESM1_ESM.docx]

**Supplementary Information**

**Supplementary Methods**

## hiPSC culture and maintenance

The hiPSC line used in the study was derived and cultured on mitotically inactivated mouse embryonic fibroblasts (MEFs, 26000 cells/cm2; CellSystems GmbH, Troisdorf, Germany) at 37°C in 5% CO2. The culture medium consist of KnockOut Dulbecco’s modified Eagle medium (KO-DMEM) supplemented with 20% KnockOut Serum Replacement (Ko-SR), 2 mM GlutaMAX, 0.1 mM 2-mercaptoethanol (2-ME) (all from Gibco, Thermo Fisher Scientific, Massachusetts, USA), 1% nonessential amino acids (NEAA) and 50 U/ml penicillin/streptomycin (Lonza, Switzerland). Medium was also supplemented with 4 ng/ml human basic fibroblast growth factor (bFGF; PeproTech, Inc. New Jersey, USA). The hiPSCs were adapted to feeder-free condition on precoated plates with LDEV-free hESC-qualified reduced growth factor basement membrane matrix Geltrex (1:100 dilution, Gibco, Thermo Fisher Scientific) in mTeSR1 medium (STEMCELL Technologies, Vancouver, Canada). Cells were passaged using versene (Gibco, Thermo Fisher Scientific) and were regularly checked for mycoplasma contamination.

## Design and Construction of CRISPR-Cas9 vectors

The CRISPR knock-in strategy for the generation of reporter line was designed on a plasmid-based system employing hCas9 (Addgene #41815) (Mali et al., 2013). Potential Cas9 target sites flanking the stop codon of the target gene MYH6 were identified using an online CRISPR design tool (crispr.mit.edu) (Ran et al., 2013). Four sgRNA were selected and cloned into a U6-driven gRNA expression vector (Addgene #41824) as previously described (Byrne et al., 2014; Mali et al., 2013). The level of activity of each sgRNA was validated by T7EI assay in HEK cells and the best guide RNA (GCAGCA AAAAATGCACGATGAGG) was selected for the MYH6-mScarlet hiPSC reporter line generation. For the construction of target vector, the eGFP sequence in the OCT4-2A-eGFP-PGK-Puro plasmid (Addgene#31938) (Hockemeyer et al., 2011) was replaced with the mScarlet sequence from pmScarlet-i_C1 (Addgene #85044) (Bindels et al., 2017) by SOE-PCR after introducing a SalI site after the eGFP stop codon by quick change mutagenesis. The 5' and 3' MYH6 homology arms, (867 base pairs) and (1,001 base pairs) respectively, were amplified from hiPSC genomic DNA using the following primers

5'H forward- TATACCTGCAGGCACAGGGCAGTGGGGAAGACATCTGG

5'H reverse - TATAGCTAGCCTCTTCATCGTGCATTTTTTGCTGC

3'H forward- ATATGGCGCGCCCACTGCCTCGGGAACCTCACTCTTGC

3'H reverse- TATAGGCCGGCCGCACAGTCCATTCTCATGGTCTCAG

The 5' and 3' homology arms were subcloned into the 2A-mScarlet-PGK-Puro plasmid using Sda 1-Nhe1 and Sgs1-Fse1 respectively. All the plasmids were isolated using an endotoxin-free Midiprep kit (QIAGEN, Hilden, Germany) and were verified by sequencing prior to genome targeting.

## Characterization of D103-4 hiPSC reporter line

The pluripotency of the genome edited cardiac reporter line was verified by PCR and immunocytochemistry as described before (Ojala et al., 2012). The presence of pluripotency genes Nanog, SOX2, REX1, OCT4, and c-MYC and the absence of virally imported exogenes (OCT4, SOX2, c-MYC, and KLF4) were confirmed by RT-PCR. GAPDH was used as an endogenous control. The primer sequences for pluripotency genes and virally imported exogenes are presented in Table S4. The expression of pluripotency markers (Nanog, OCT-3/4, SOX2, SSEA-4, TRA 1-60 and TRA 1-81) were analyzed by staining the paraformaldehyde (PFA 4%, MERK, New Jersey, USA) fixed D103-4 hiPSC colonies with the primary antibodies and the respective secondary antibodies as listed in Table S5.

## Cardiomyocyte differentiation

The differentiation of D103-4 hiPSCs reporter line into CMs was mostly performed using small molecule (SM-D) method as previously described (Lian et al., 2013). Briefly, the differentiation was initiated, when the hiPSC-culture was 100% confluent, by changing the mTeSR1 medium to insulin-free RPMI/B27 (Thermo Fisher Scientific, Gibco) medium containing 8 μM CHIR99021 (tebu-bio, BPS Bioscience, France) denoted as day 0. After 24 hrs, CHIR99021 was withdrawn by changing the medium to insulin-free RPMI/B27 (day 1). Half of the cell culture medium was replaced with fresh insulin-free RPMI/B27 medium every other day. On day 3, 5 μM IWP4 (Tocris Bioscience) was added to the medium for 48 hr. On day 5 and 7, the medium was refreshed and from day 10 forward, half of the medium was replaced with fresh RPMI/B27 medium with insulin (Thermo Fisher Scientific, Gibco) three times a week. The D103-4 hiPSCs reporter line was also differentiated into CMs by coculturing with mouse visceral endodermal-like cells (END-2) (Hubrecht Institute, Utrecht, Netherlands) (Mummery et al., 2003) and by a modified EB method with Activin A and BMP-4 (Karakikes et al., 2014; Prajapati et al., 2021).

## Electrophysiology characterization

## Patch Clamp recording

Current-clamp recordings were digitally sampled at 20 kHz and filtered at 2 kHz using low pass Bessel filter on recording amplifier. The extracellular solution contained (in mM) 143 NaCl, 4.8 KCl, 1.8 CaCl_2_, 1.2 MgCl_2_, 5 glucose, and 10 4-(2-hydroxyethyl)-1-piperazineethanesulfonic acid (HEPES) (pH was adjusted to 7.4 with NaOH). The preheated extracellular solution to 36±1°C was continuously perfused. The patch electrodes had tip resistance of 2.0–3.0 MΩ and contained the following intracellular solution (in mM): 132 KMeSO_4_, 20 KCl, 1 MgCl_2_, 4 ethylene glycol-bis (β-aminoethyl ether)-N,N,N0,N0-tetraacetic acid (EGTA) and 1 CaCl_2_ (pH was adjusted to 7.2 with KOH). From the recorded APs, beats per minute (BPM), AP duration at 50% and 90% repolarization (APD50 and APD90), AP amplitude (APA), maximal upstroke velocity (dV/dtMax) and maximum diastolic potential (MDP) were analyzed by using custom made software in Origin 9.1 (OriginLab Corp., Northampton, USA). Only ventricular-like APs, which were characterized by APD90/APD50 < 1.3 and APA > 90 mV were used for comparison. To stimulate the CMs, negative currents were injected to hyperpolarize CMs around -70 mV from where 2-4 ms depolarizing currents at 1 Hz were applied.

The amplifier was switched into voltage clamp mode to record the ionic currents from hiPSC-CMs by using perforated whole cell method maintaining the same extracellular and intracellular solutions. The ionic currents were measured at 36±1°C. The holding potential was −40 mV to inactivate the Na^+^ channels. The Ca^2+^ current (ICa) was measured with a depolarizing potential from −50 mV to 80 mV of 300 ms duration with 10 mV increments. In addition, 2 mM 4-AP and 1 μM E-4031 were used in the extracellular solution to block the transient outward K^+^ current and rapid delayed rectifying K^+^ current (IKr) respectively. Furthermore, IKr current was calculated as 1 μM E-4031 sensitive current in the presence of 5 μM Nimodipine to block ICa. For this, depolarizing potential from -40 to 40 mV of 4 sec duration with 20 mV increment protocol was used. The Peak and tail IKr were calculated from the end of the test pulse and peak of the tail current respectively. Recorded ionic currents were divided by cell capacitances and presented as pA/pF.

## Calcium imaging

Dissociated spontaneously beating CMs on a coverslip were loaded with 4 μM Fluo-4 AM (Life Technologies Ltd). CMs were continuously perfused with 37 °C HEPES based medium during Ca^2+^ imaging measurements consisting of (in mM) 137 NaCl, 5 KCl, 0.44 KH2PO_4_, 20 HEPES, 4.2 NaHCO_3_, 5 D-glucose, 2 CaCl_2_, 1.2 MgCl_2_ and 1 Na-pyruvate (pH was adjusted to 7.4 with NaOH). Ca^2+^ kinetics was imaged with Axio Observer.A1 microscope with Objective Fluar 20x/0.75 M27 (both Carl Zeiss Microscopy GmbH, Göttingen, Germany). Images were acquired with an ANDOR iXon3 885 EM-CCD camera (Andor Technology, Belfast, Northern Ireland) synchronized with Lambda DG-4 Plus (Sutter Instrument, California, USA) wavelength switcher, ZEISS Filter set 46 (Carl Zeiss Microscopy GmbH, Göttingen, Germany) and ZEN 2 blue edition software (Carl Zeiss Microscopy GmbH, Göttingen, Germany). For Ca2+ analysis, regions of interest were selected for spontaneously beating cells and background noise was subtracted before further data processing. All the recordings were at least 30sec long with a sampling rate was 20 to 30 ms.

**Supplementary Figures**


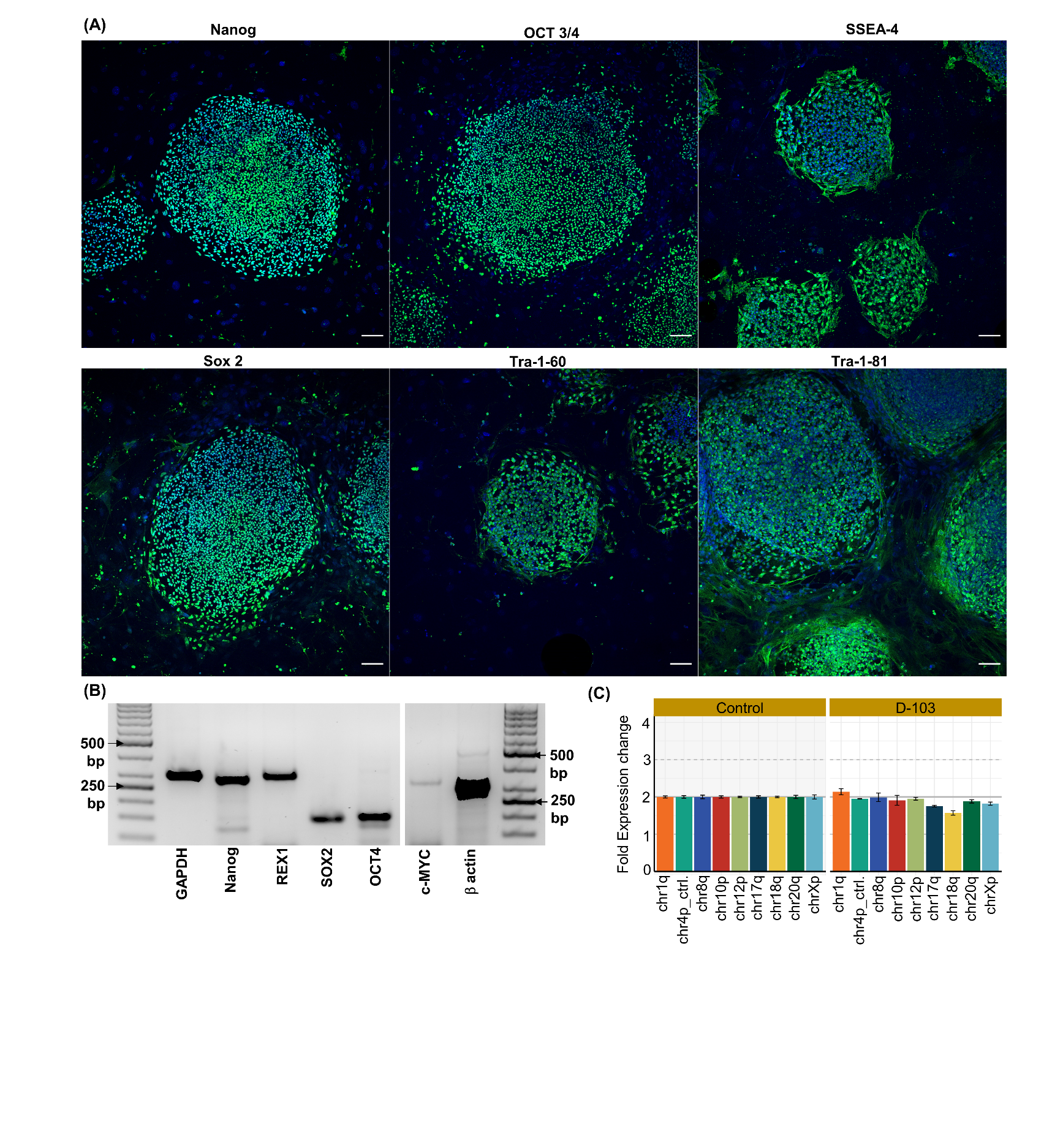


**Fig. S1.** **Expression of pluripotency markers and karyotype analysis of D103-4 hiPSC cardiac reporter line. a.** Representative images of hiPSC colonies expressing Nanog, OCT-3/4 SSEA-4, Sox-2, TRA-1-60 and TRA-1-81 is shown. Scale bars are 100 μM. **b**. hiPSCs expressed endogenous Nanog (287 bp), REX1 (306 bp), SOX2 (151 bp), OCT4 (144 bp), and c-MYC (328 bp) and GAPDH (302 bp) and βactin was used as a housekeeping control. **c.** The D103-4 hiPSC line was karyotypically normal using hPSC Genetic Analysis kit.

**Day 1**

**Day 2**

**Day 3**

**Day 4**


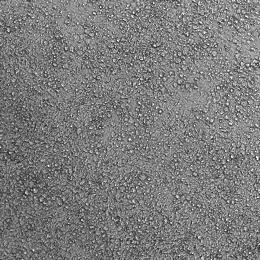

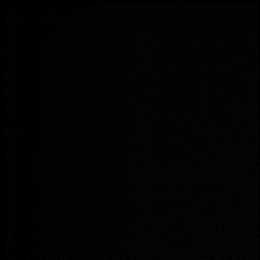

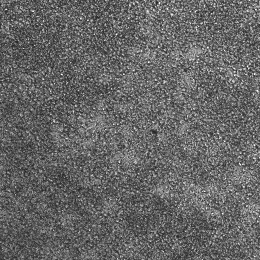

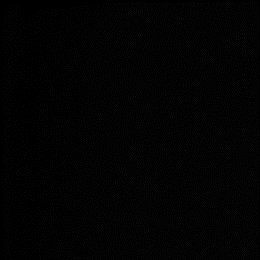

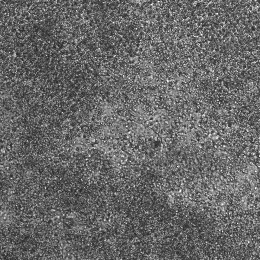

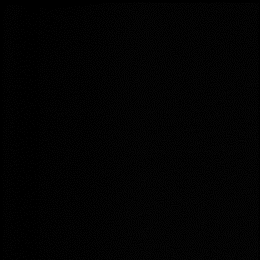

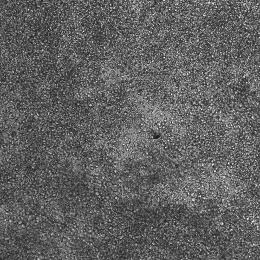

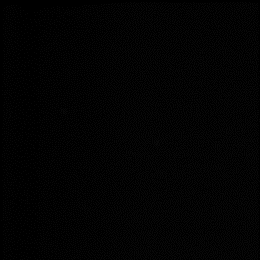


**Fig. S2.** **MYH6-mScarlet reporter expression profile monitored during CM differentiation course (day 1 to day 4) by a time-lapse imaging system**, Scale bar is 100 μm. From day 1 to 4, there is no mScarlet expression (down panel images).


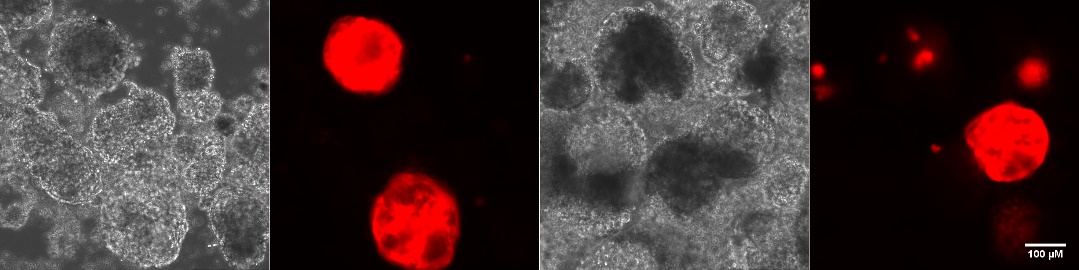

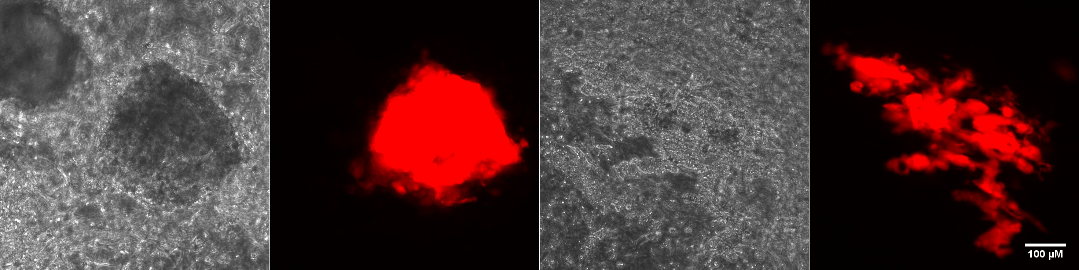


**END2 Method**

**EB Method**

**Fig. S3.** **Differentiation of** **D103-4 reporter line using END-2 co-culture method and modified EB method. A.** Representative images of mScarlet expression in the CMs during END2 and modified EB differentiation methods, validating the mScarlet reporter activity in D013-4 hiPSC line.


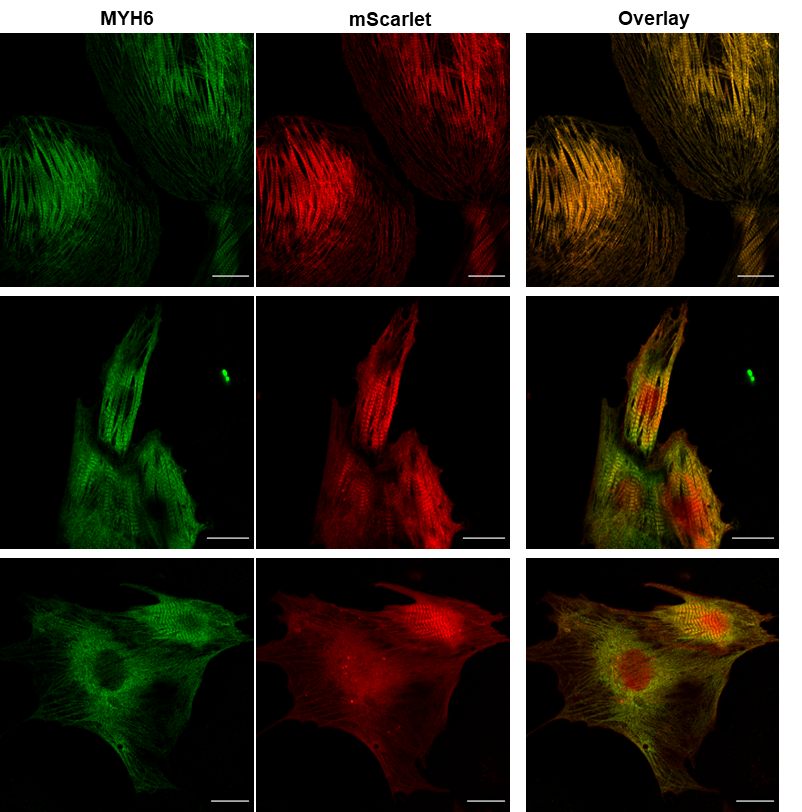


**Fig. S4.** **Immunostaining of** **D103-4 reporter CMs with anti-MYH6 that shows the co-localization of the mScarlet reporter fluorescence with MYH6 staining.** In the overlay images, most of the mScarlet exist as MYH6-mScarlet fusion protein staining the sarcomere. Scale bar is 100 μm


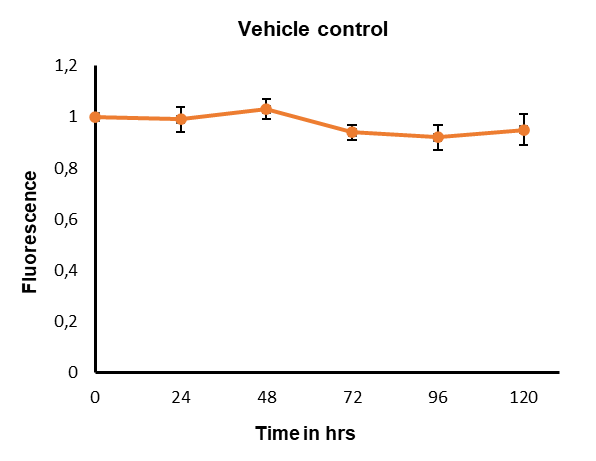


**Fig. S5.** **Fluorescence plot for the vehicle control during 120 hrs.** The fluorescence intensity at each time point was normalized to the values obtained at the 0hr of the vehicle control treatment. Data are presented as mean ± SD.

**
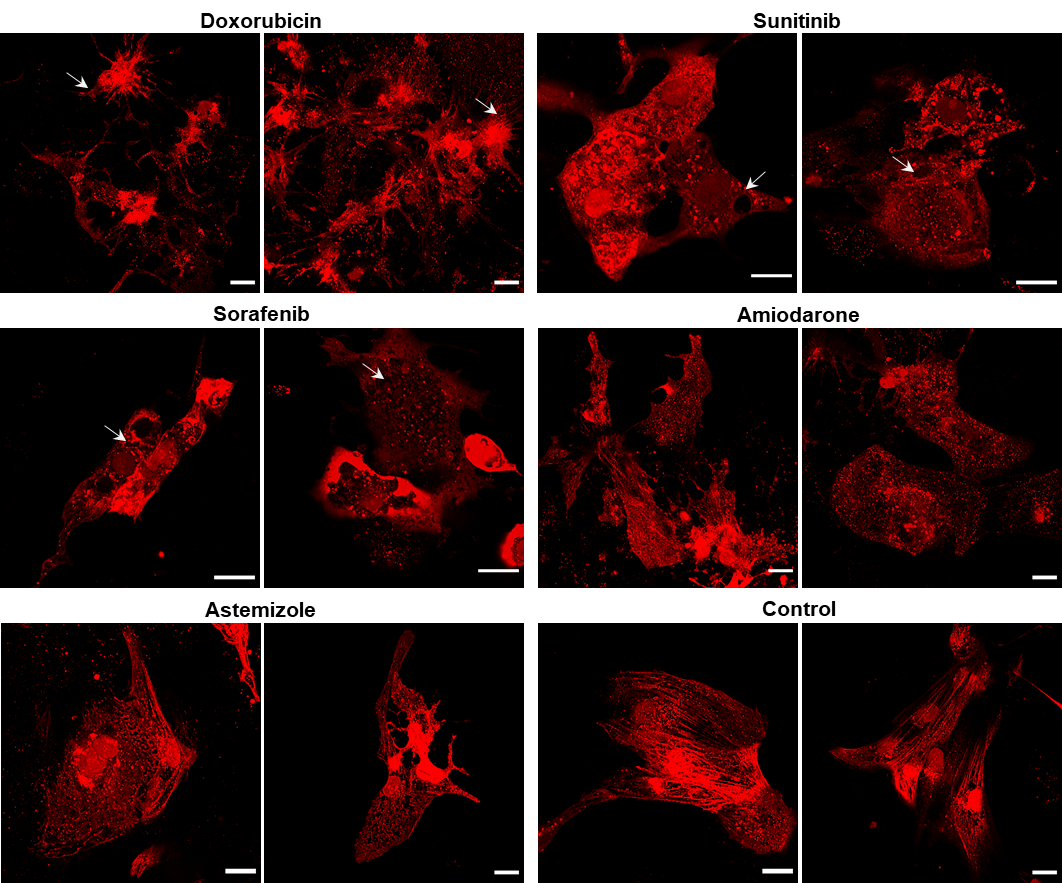
**

**Fig S6**. **Confocal images of the reporter CMs showing structural cardiotoxicity after 72hrs of respective drug treatment**. CMs were treated with drug dose that depletes 100% ATP level in 120 hrs (Doxorubicin 5 μM, Sunitinib 20 μM, Sorafenib 60 μM, Amiodarone 60 μM, Astemizole 20 μM). At 72 hrs, drug treated CMs were observed for drug specific phenotypical changes. Myofibril loss and sarcomere disarray altering the cardiomyocyte architecture can be detected by mScarlet fluorescence without immunostaining the CMs for structural markers, Scale bar is 20 μM.


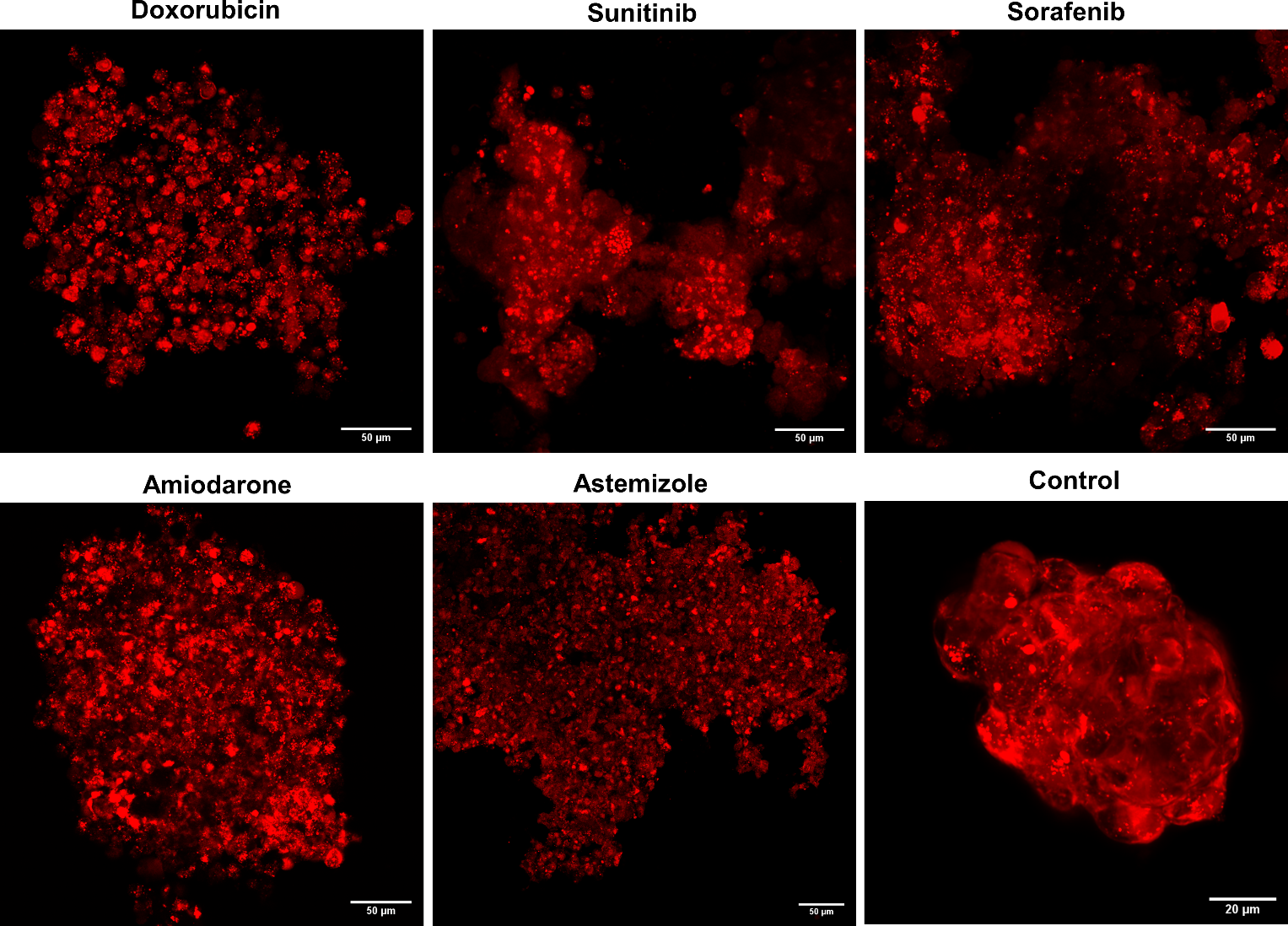


**Fig S7**. **Drug toxicity assay in the EB clusters of reporter hiPSC-CMs showing cardiotoxicity in 3D culture environment.** CM clusters were treated with the same drug dose as in the 2D culture (Doxorubicin 5 μM, Sunitinib 20 μM, Sorafenib 60 μM, Amiodarone 60 μM, Astemizole 20 μM) to visualize the phenotypical changes in the 3D culture conditions. Confocal microscopy of CM clusters after 72 hrs of drug treatment, showing apoptotic/necrotic CMs and disrupted cell-cell contact compared to the vehicle control.

**Supplementary Tables**

**Table S1.** Action potential parameters from spontaneous beating WT and D103-4. n represents the number of cells. Data are presented as mean±sem. ns represents non significance as calculated by student t test. BR: Beats Rate, APD50/90: Action Potential Duration at 50% and 90% repolarization, APA: Action Potential Amplitude, MDP: Maximum Diastolic Potential, dV/dtMax: Maximum Upstroke Velocity.

|  | **WT (n=13)** | **D103-4 (n=17)** | **Statistical Significance** |
| --- | --- | --- | --- |
| **BR (bpm)** | 42.4±5.3 | 51.0±3.4 | ns |
| **APD50 (ms)** | 203.3±21.5 | 200.9±15.0 | ns |
| **APD90 (ms)** | 254.7±24.8 | 247.2±17.8 | ns |
| **APA (mV)** | 115.0±2.0 | 114.5±2.0 | ns |
| **MDP (mV)** | -71.7±1.6 | -69.2±1.6 | ns |
| **dV/dT_Max_ (V/s)** | 32.9±5.8 | 29.9 | ns |

**Table S2.** Calcium transient parameters from spontaneous beating WT and D103-4. n represents the number of cells. Data are presented as mean±sem. ns represents non significance as calculated by student t test. Freq: frequency, TPeak: Time to reach peak, T90: Time to decay to 90%.

|  | **WT (n=18)** | **D103-4 (n=18)** | **Statistical Significance** |
| --- | --- | --- | --- |
| **Freq (Hz)** | 0.6±0.1 | 0.5±0.1 | ns |
| **TPeak (ms)** | 299.5±42.5 | 417±49.6 | ns |
| **T90 (ms)** | 750.1±76.4 | 959.9±87.8 | ns |

**Table S3:** Delayed rectifying potassium current densities recorded from WT and D103-4. mV represents the membrane potential. n represents the number of cells used. Data are presented as mean±sem. ns represents the not significance as calculated student t-test.

| **mV** | **WT (n=7)** | **D103-4 (n=7)** | **Statistical Significance** |
| --- | --- | --- | --- |
| Peak current | | | |
| **-40** | 0.3±0.1 | 0.3±0.2 | ns |
| **-20** | 1.2±0.3 | 1.3±0.2 | ns |
| **0** | 1.9±0.6 | 2.0±0.3 | ns |
| **20** | 1.5±0.5 | 1.5±0.3 | ns |
| **40** | 1.1±0.3 | 1.2±0.3 | ns |
| Tail current | | | |
| **-40** | 0.3±0.1 | 0.3±0.2 | ns |
| **-20** | 1.2±0.2 | 1.3±0.3 | ns |
| **0** | 2.0±0.4 | 2.1±0.3 | ns |
| **20** | 2.1±0.5 | 2.1±0.3 | ns |
| **40** | 2.2±0.5 | 2.3±0.5 | ns |

**Table S4:** Primers used for the pluripotency analysis

| **Gene** | **Target** | **Primer sequence** | **Size-bp** |
| --- | --- | --- | --- |
| PAX-6 (Paired box 6) | Ectoderm | **F:** AACAGACACAGCCCT  **R:** CGGGAACTTGAACTG | 274 |
| SOX-1 (SRY- sex determining region Y-box 1) | Ectoderm | **F:** CACAACTCGGAGATC  **R:** GTCCTTCTTGAGCAG | 171 |
| AFP (Alpha-fetoprotein) | Endoderm | **F:** GCTGGATTGTCTGCA  **R:** TCCCCTGAAAAT | 216 |
| SOX-17 (SRY (sex determining region Y)-box 17) | Endoderm | **F:** CGCACGGAATTTGAA  **R:** CACACGTCAGGATAG | 166 |
| α -cardiac actin (Actin, alpha, cardiac muscle 1) | Mesoderm | **F:** GGAGTTATGGTGGGTATGGGTC  **R:** AGTGGTGACAAAGGAGTAGCC | 486 |
| KDR (Kinase insert domain receptor) | Mesoderm | **F:** GTGACCAACATGGAGTCGTG  **R:** TGCTTCACAGAAGACCATGC | 218 |
| Nanog | Pluripotency | **F:** CAGCCCCGATTCTTCCACCAGTCCC  **R:** CGGAAGATTCCCAGTCGGGTTCAC | 287 |
| REX1 (reduced expression 1) | Pluripotency | **F**: CAGATCCTAAACAGCTCGCAGAAT  **R**: GCGTACGCAAATTAAAGTCCAGA | 306 |
| SOX-2 (sex determining region Y-box 2) | Pluripotency | **F**: GGGAAATGGGAGGGGTGCAAAAG  **R**: TGCGTGAGTGTGGATGGGATTGG TG | 151 |
| OCT4 | Pluripotency | **F**:GACAGGGGGAGGGGAGGAGCTAGG  **R**:CTTCCCTCCAACCAGTTGCCCCAAA | 144 |
| c-MYC | Pluripotency | **F:** GCGTCCTGGGAAGGGAGATCCGGG  **R:** TTGAGGGGCATCGTCGCGGGAGGC TG | 328 |
| β-actin (Actin, beta) | Housekeeping control | **F:** GTCTTCCCCTCCATC  **R:** GGGGTGTTGAAGGTC | 302 |

**Table S5:** Antibodies used for the immunostaining of pluripotency markers

| **Antibody** | **Origin** | **Dilution** | **Catalogue number** | **Manufacturer** |
| --- | --- | --- | --- | --- |
| OCT-3/4 | goat | 1:400 | AF1759 | R&D Systems |
| Nanog | goat | 1:200 | AF1997 | R&D Systems |
| SSEA-4 | mouse | 1:200 | sc-21704 | Santa Cruz Biotechnology |
| SOX2 | goat | 1:200 | sc-17319 | Santa Cruz Biotechnology |
| TRA-1-60 | mouse | 1:200 | MAB4360 | Millipore |
| TRA-1-81 | mouse | 1:200 | MAB4381 | Millipore |

**Table S6:** TaqMan assays used in the qRT-PCR protocol.

| **Gene** | **Description** | **Function** | **TaqMan assay ID** |
| --- | --- | --- | --- |
| TNNT2 | Cardiac type troponin T2 | Sarcomeric gene | Hs00165960_m1 |
| TPM1 | α-tropomyosin | Sarcomeric gene | Hs00165966_m1 |
| ACTN2 | α-actinin 2 | Sarcomeric gene | Hs00153809_m1 |
| TTN | Titin | Sarcomeric gene | Hs00399225_m1 |
| MYBPC3 | Myosin binding protein C, cardiac | Sarcomeric gene | Hs00165232_m1 |
| MYH6 | Myosin heavy chain 6 | Sarcomeric gene | Hs01101425_m1 |
| MYH7 | Myosin heavy chain 7 | Sarcomeric gene | Hs01110632_m1 |
| MYL2 | Myosin regulatory light chain 2 | Sarcomeric gene | Hs00166405_m1 |
| MYL7 | Myosin regulatory light chain 7 | Sarcomeric gene | Hs01085598_g1 |
| GJA1 | Gap junction alpha-1 protein | Connexin 43 | Hs00748445_s1 |
| ATP2A2 | ATPase, calcium transporting, cardiac muscle, slow twitch 2/ SERCA2a | Calcium ATPase | Hs00544877_m1 |
| CASQ2 | Calsequestrin | Calcium binding protein | Hs00154286_m1 |
| RYR2 | Ryanodine receptor 2, cardiac | Ryanodine receptor | Hs00892883_m1 |
| SCN5A | Voltage-gated sodium channel, V type, alpha subunit | Sodium channel | Hs00165693_m1 |
| KCNJ2 | Potassium voltage-gated channel subfamily J member 2 | Potassium channel | Hs01876357_s1 |
| HCN4 | Hyperpolarization activated cyclic nucleotide-gated potassium channel 4 | Potassium channel | Hs00975492_m1 |
| GAPDH | Glyceraldehyde-3-phosphate dehydrogenase | Housekeeping gene | Hs02758991_g1 |
| EEF1A1; EE+ | Eukaryotic translation elongation factor 1 alpha 1 | Housekeeping gene | Hs00265885_g1 |

**Movie Captions**

**Movie 1**: Single/selective plane illumination microscopy (SPIM) showing the 3D structural organization of the reporter CMs within a cluster

**Movie 2**: Shutdown of mScarlet fluorescence in the hiPSC-CMs treated with 5 μM Doxorubicin for 120 hrs

**Movie 3: Mscarlet** fluorescence in the vehicle control hiPSC-CMs.

**Movie 4**: Time lapse imaging of reporter CMs treated with 5 μM Doxorubicin

**Movie 5**: Time lapse imaging of reporter CMs treated with 20 μM Sunitinib

**Movie 6**: Time lapse imaging of reporter CMs treated with 60 μM Sorafenib

**Movie 7**: Time lapse imaging of reporter CMs treated with 60 μM Amiodarone

**Movie 8**: Time lapse imaging of reporter CMs treated with 20 μM Astemizole

**Movie 9**: Time lapse imaging of reporter CMs treated with vehicle control
